# Supplementary material for: Major features of parasite adaptation revealed by genomes of Plasmodium falciparum population samples archived for over 50 years
Source: Commun Biol. 2026 Jan 12;9:183. doi: 10.1038/s42003-025-09460-3 (PMC12880971; doi:10.1038/s42003-025-09460-3)
Supplement: Supplementary file 2 — Supplementary Figs. [file 42003_2025_9460_MOESM2_ESM.pdf]

Major features of parasite adaptation revealed by genomes of *Plasmodium falciparum* population samples archived for over 50 years

Supplementary Information

Supplementary Figures included here

Supplementary Tables are accessible separately as EXCEL files

Alfred Amambua-Ngwa [1,2,3*], Mouhamadou Fadel Diop [1], Christopher J. Drakeley [2], Umberto d'Alessandro [1], Dominic P. Kwiatkowski [3#], David J. Conway [2*]

[1]MRC Unit The Gambia at London School of Hygiene and Tropical Medicine, Banjul, The Gambia

[2] Department of Infection Biology, London School of Hygiene and Tropical Medicine, London, UK

[3] Wellcome Sanger Institute, Hinxton, UK

Died in April 2023

* Correspondence Emails: alfred.ngwa@lshtm.ac.uk, david.conway@lshtm.ac.uk

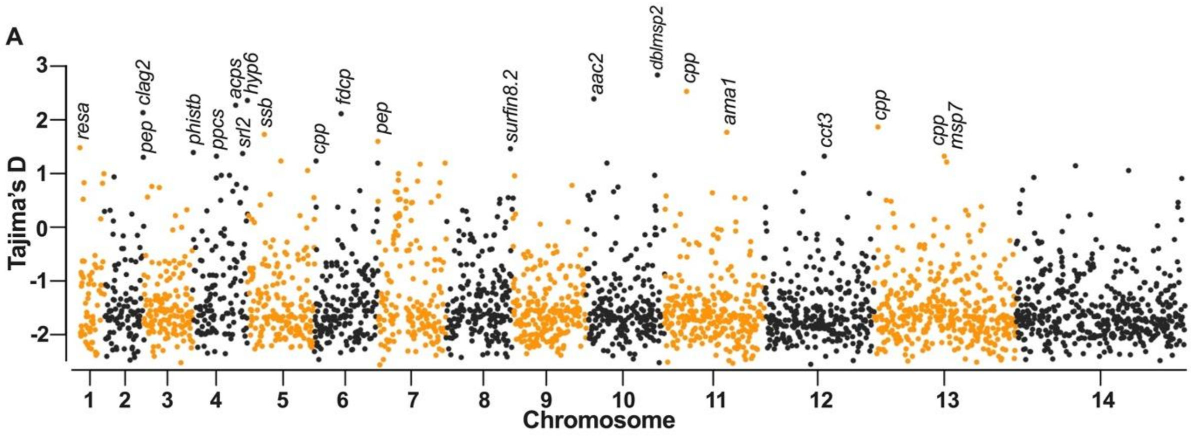

B

Gene Symbol	Gene ID	Gene Description	N	S	Tajima's D
resa	PF3D7_0102200	ring-infected erythrocyte surface antigen	48	6	1.49
clag2	PF3D7_0220800	cytoadherence linked asexual protein 2	38	42	2.15
pep	PF3D7_0221000	Plasmodium exported protein, unknown function	49	23	1.31
pfd80	PF3D7_0401800	Plasmodium exported protein, unknown function	51	5	1.40
ppcs	PF3D7_0412300	phosphopantothenoylcysteine synthetase, putative	49	10	1.33
acps	PF3D7_0420200	holo-[acyl-carrier-protein] synthase, putative	50	15	2.29
sr12	PF3D7_0422800	serpentine receptor 12, putative	46	10	1.38
hyp6	PF3D7_0425100	Plasmodium exported protein , unknown function	47	27	2.38
ssb	PF3D7_0508800	single-stranded DNA-binding protein	52	3	1.74
cpp	PF3D7_0602700	conserved Plasmodium protein, unknown function	52	8	1.24
fdcp	PF3D7_0614100	filamin domain-containing protein, putative	50	6	2.13
pep	PF3D7_0701900	Plasmodium exported protein, unknown function	48	55	1.61
surfin8.2	PF3D7_0830800	surface-associated interspersed protein 8.2	26	141	1.47
aac2	PF3D7_1004800	ADP,ATP carrier protein 2	51	9	2.41
dblmsp2	PF3D7_1036300	duffy binding-like merozoite surface protein 2	45	23	2.86
cpp	PF3D7_1113600	conserved Plasmodium protein, unknown function	50	5	2.55
ama1	PF3D7_1133400	apical membrane antigen 1	43	45	1.78
cct3	PF3D7_1229500	T-complex protein 1 subunit gamma	54	4	1.33
cpp	PF3D7_1302900	conserved protein, unknown function	47	17	1.88
msp7	PF3D7_1335100	merozoite surface protein 7	45	21	1.33

Supplementary Fig. 1. Genome-wide scan for *P. falciparum* genes with more intermediate allele frequencies than expected indicating balancing selection in the sampled population from 1966 – 1971 in The Gambia. **A.** Tajima's D values are plotted for each genes containing at least 3 SNPs, and the genes with the top 20 highest values are labelled as these indicate the most exceptional patterns of intermediate allele frequencies. **B.** Description and summary of diversity for the genes having the top 20 values of Tajima's D index. N indicates the number of samples with sequences contributing to the analysis for each gene. S is the number of polymorphic sites. A list of results for all genes in the core genome with at least 3 SNPs is presented in Supplementary Table 4.

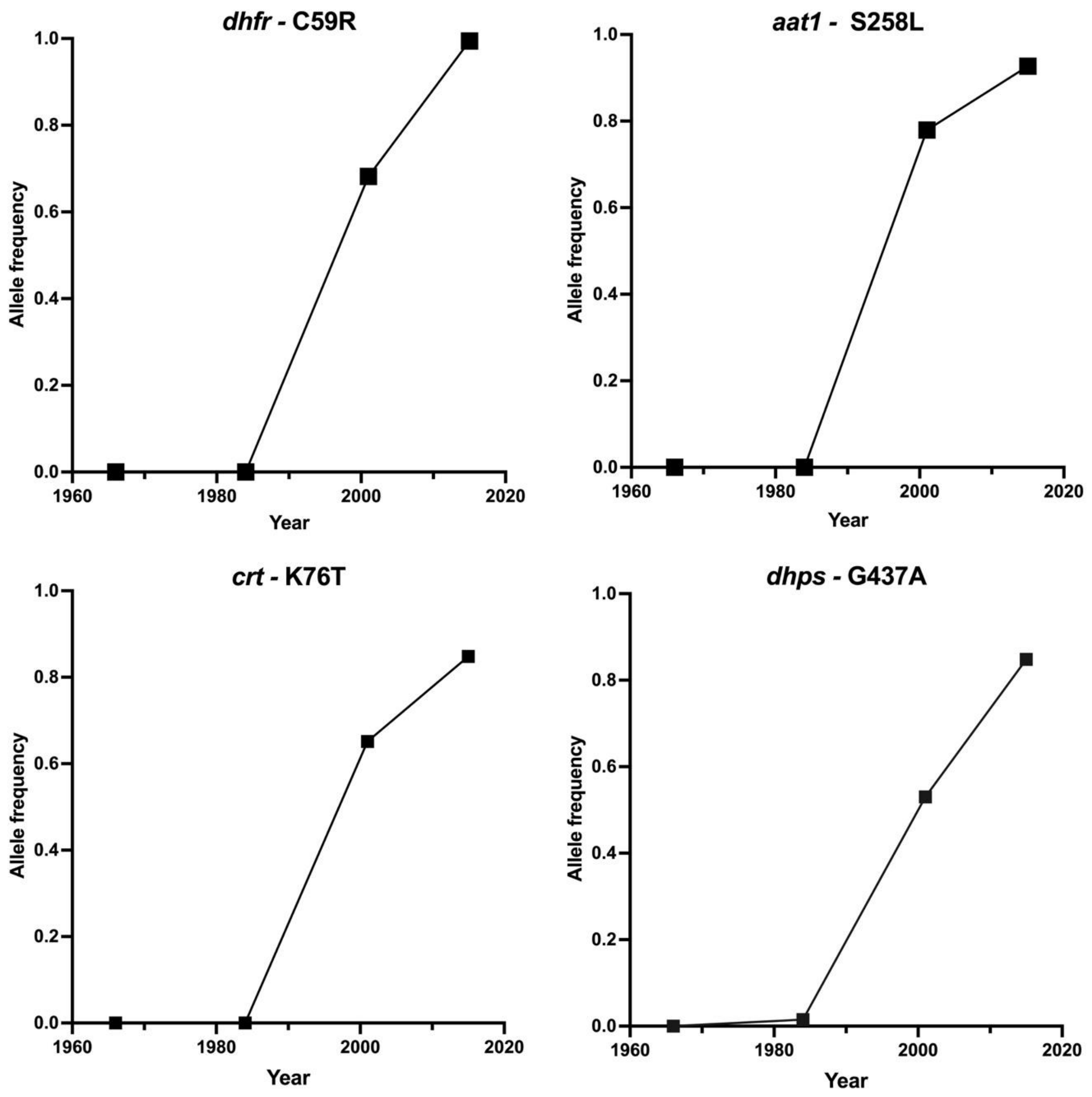

Supplementary Fig. 2. Temporal allele frequency changes over a period of almost 50 years in The Gambia for known drug resistance associated mutations in dihydrofolate synthetase (*dhfr*), amino acid transporter (*aat1*), chloroquine resistance transporter (*crt*) and dihydropteroate synthetase (*dhps*). A full list of non-synonymous SNPs with significant allele frequency change over time is presented in Supplementary Table 9.

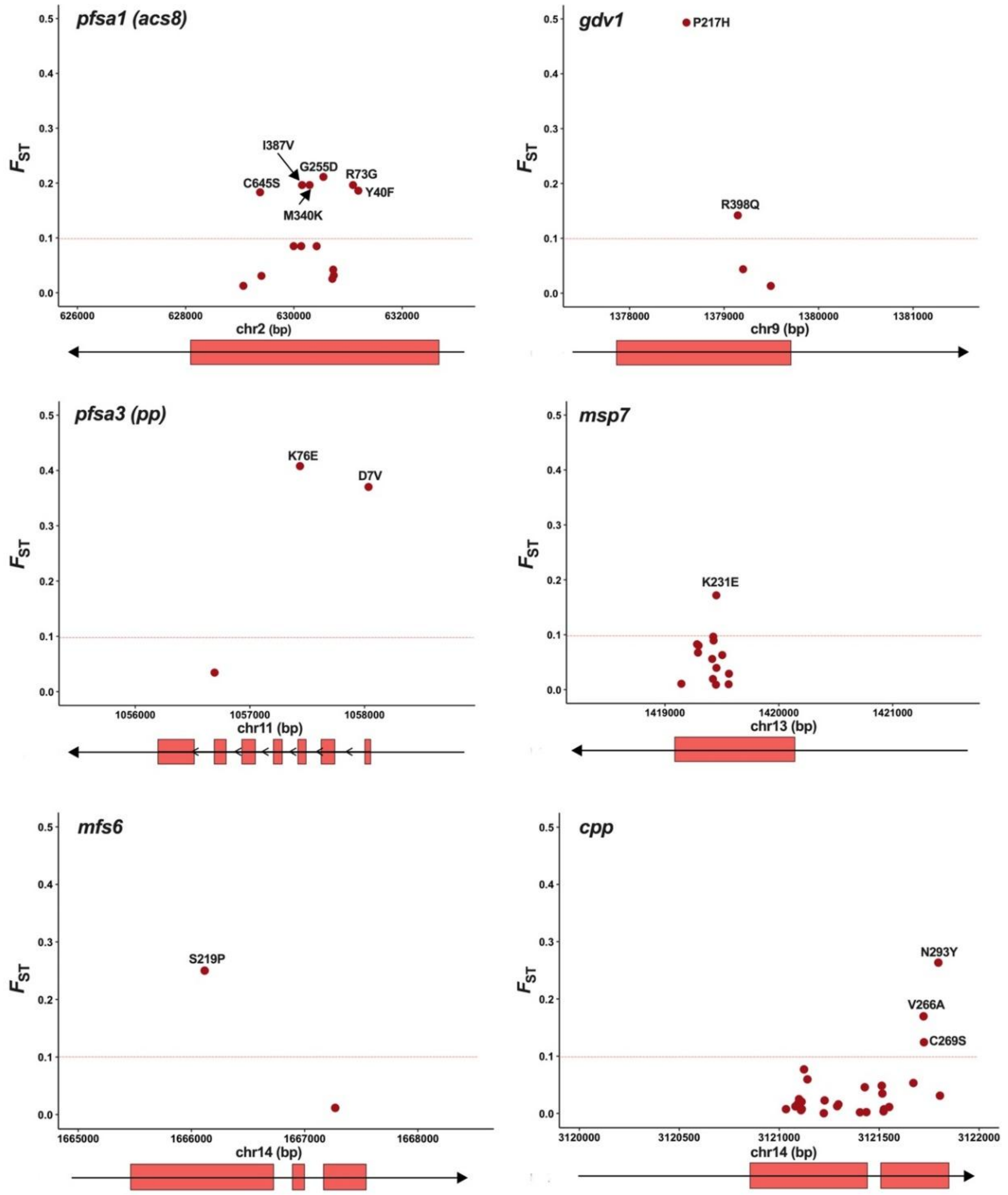

Supplementary Fig. 3. Zoomed-in view of six *P. falciparum* gene loci not associated with drug resistance that had highly differentiated SNP allele frequencies in The Gambia in a comparison between 1966-1971 and 2015 (temporal F_{ST} fixation indices > 0.1). These are the six loci shown boxed on Fig. 4. Each panel presents F_{ST} values for non-synonymous SNPs plotted against their respective chromosomal (chr) positions, and the gene abbreviation labelled at the top left. Codon positions and amino acid alleles for non-synonymous variants with F_{ST} > 0.1 are indicated. The gene exons and the direction of transcription in relation to the genomic coordinates are shown beneath each plot. The gene abbreviations are: *pfsa1*, *P. falciparum* sickle-associated locus 1 coding for acetyl coenzyme A synthetase 8 (*acs8*); *gdv1*, gametocyte development protein1; *pfsa3*, *P. falciparum* sickle-associated locus 3 coding for a putative protein phosphatase (*pp*); *msp7*, merozoite surface protein 7; *mfs6*, putative major facilitator superfamily domain-containing protein; *cpp*, conserved *Plasmodium* protein with gene ontology prediction as an integral membrane component.

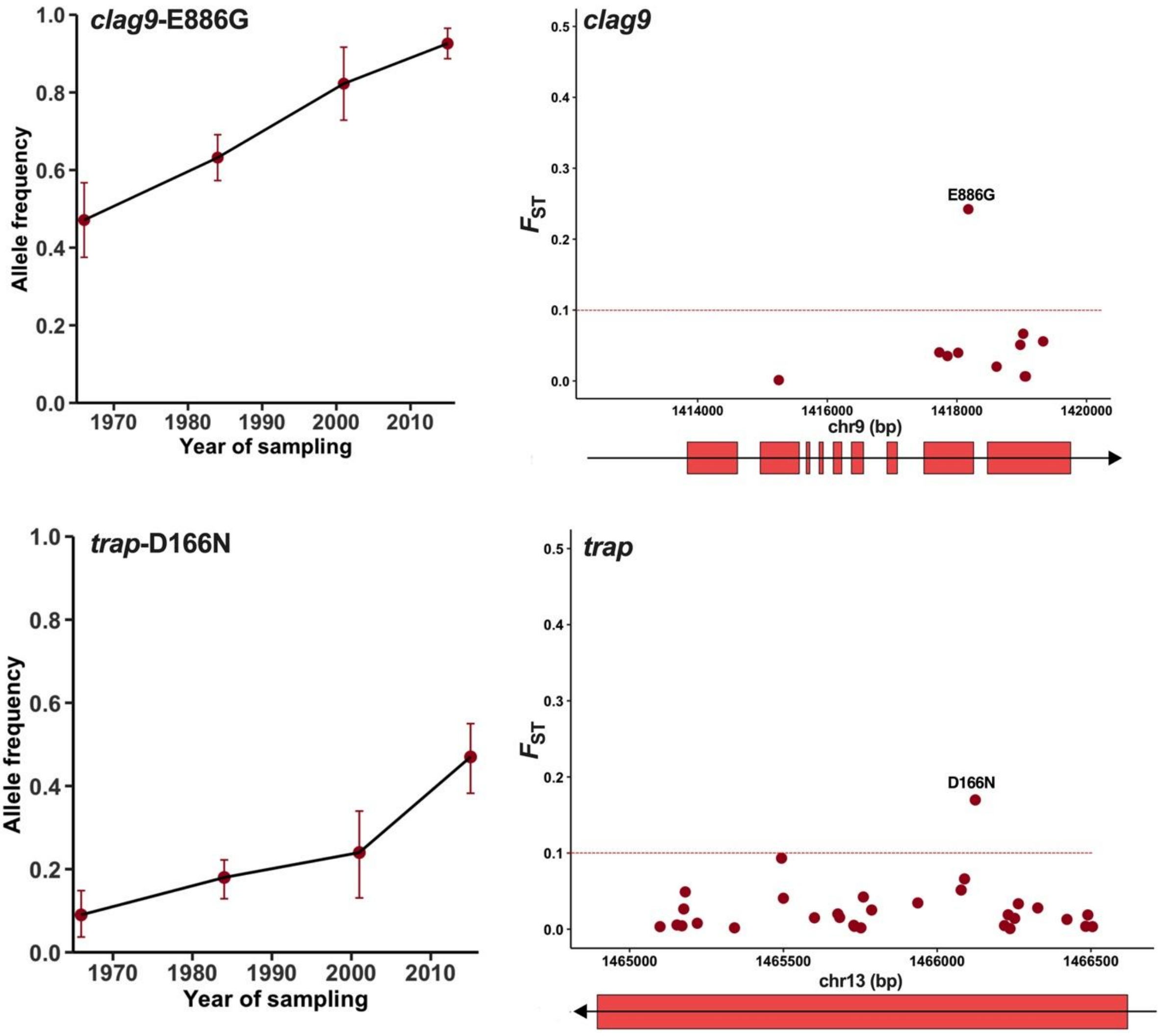

Supplementary Fig. 4. Allele frequency changes over time for SNPs in two genes closely linked with others showing significant changes in Fig. 4. The top row shows allele frequencies at different times from 1966-71 (plotted by with the median sample year 1968) onwards for variant E886G in *clag9* adjacent to a plot of the F_{ST} values of all coding SNPs in the gene on chromosome 9. Exons depicted as red bars and arrows show the coding frame. The second row shows equivalent plots for the *trap* gene on chromosome 13, with allele frequencies showing frequency changes of the most temporally differentiated variant D166N.
