# Supplementary material for: Major features of parasite adaptation revealed by genomes of Plasmodium falciparum population samples archived for over 50 years
Source: Commun Biol. 2026 Jan 12;9:183. doi: 10.1038/s42003-025-09460-3 (PMC12880971; doi:10.1038/s42003-025-09460-3)
Supplement: Supplementary file 3 — Description of Additional Supplementary Files [file 42003_2025_9460_MOESM3_ESM.pdf]

Description of Additional Supplementary Files

File name- Supplementary Data1.

Description: Metadata and P. falciparum sequence coverage for 60 parasite-positive placental blood samples collected between 1966 and 1971 in the greater Banjul area of The Gambia. Source data behind Figures 1-4.

File name- Supplementary Data 2.

Description: Genome-wide listing of BetaScore indices for P. falciparum SNPs in the 1966-1971 population analysis based on 54 infections with high coverage sequences. Source data behind Figure 1.

File name- Supplementary Data 3.

Description: Genome-wide scan of integrated haplotype score (iHS) for P. falciparum SNPs in the 1966-1971 Gambian population sample of 54 infections with high-quality sequences. Source data behind Figure 1.

File name- Supplementary Data 4.

Description: Genome-wide listing of Tajima's D values for all P. falciparum core genes with at least 3 SNPs in the 1966-1971 Gambian population.

File name- Supplementary Data 5.

Description: Genome-wide P. falciparum withininfection fixation indices (FWS) and Complexity of infection (COI) estimates for 54 archived Gambian infection samples from 1966-1971 with highquality sequences. Source data behind Figure 2.

File name- Supplementary Data 6.

Description: Metadata and P. falciparum sequence coverage for 89 infection samples from uncomplicated malaria cases in 2015 in the greater Banjul area of The Gambia. Source data behind Figures 2-4.

File name- Supplementary Data 7.

Description: Genome-wide P. falciparum withininfection fixation indices (FWS) and Complexity of infection (COI) estimates for 89 infection samples from uncomplicated malaria cases in 2015 in the greater Banjul area of The Gambia. Source data behind Figure 2.

File name- Supplementary Data 8.

Description: Genome-wide list of FST fixation indices for P. falciparum SNPs in comparison of frequencies between 1966-1971 and 2015 in The Gambia. Source data behind Figure 4.

File name- Supplementary Data 9.

Description: Allele frequencies of P. falciparum non-synonymous SNPs with values of FST >0.1 between 1966-1971 and 2015 in The Gambia. Source data behind Figure 4.
